# The Composition of Triterpene Glycosides in the Sea Cucumber *Psolus peronii*: Anticancer Activity of the Glycosides against Three Human Breast Cancer Cell Lines and Quantitative Structure–Activity Relationships (QSAR)

**DOI:** 10.3390/md22070292

**Published:** 2024-06-26

**Authors:** Alexandra Sergeevna Silchenko, Anatoly Ivanovich Kalinovsky, Sergey Anatolievich Avilov, Roman Sergeevich Popov, Ekaterina Alexandrovna Chingizova, Ekaterina Sergeevna Menchinskaya, Elena Alexandrovna Zelepuga, Kseniya Mikhailovna Tabakmakher, Vadim Georgievich Stepanov, Vladimir Ivanovich Kalinin

**Affiliations:** 1 G.B. Elyakov Pacific Institute of Bioorganic Chemistry, Far Eastern Branch of the Russian Academy of Sciences, Pr. 100-letya Vladivostoka 159, 690022 Vladivostok, Russia; kaaniv@piboc.dvo.ru (A.I.K.); avilov_sa@piboc.dvo.ru (S.A.A.); popov_rs@piboc.dvo.ru (R.S.P.); chingizova_ea@piboc.dvo.ru (E.A.C.); ekaterinamenchinskaya@gmail.com (E.S.M.); zel@piboc.dvo.ru (E.A.Z.); tabakmakher_km@piboc.dvo.ru (K.M.T.); 2 Kamchatka Branch of Pacific Institute of Geography, Far Eastern Branch of the Russian Academy of Sciences, Partizanskaya st. 6, 683000 Petropavlovsk-Kamchatsky, Russia; stepanovvadim24@gmail.com

**Keywords:** *Psolus peronii*, triterpene glycosides, psolusosides, sea cucumber, cytotoxic activity, breast cancer, QSAR

## Abstract

Eight sulfated triterpene glycosides, peronioside A (**1**) and psolusosides A (**2**), B (**3**), G (**4**), I (**5**), L (**6**), N (**7**) and P (**8**), were isolated from the sea cucumber *Psolus peronii*. Peronioside A (**1**) is a new glycoside, while compounds **2**–**8** were found previously in *Psolus fabricii*, indicating the phylogenetic and systematic closeness of these species of sea cucumbers. The activity of **1**–**8** against human erythrocytes and their cytotoxicity against the breast cancer cell lines MCF-7, T-47D and triple-negative MDA-MB-231 were tested. The most active against cancer cell compounds, psolusosides A (**2**) and L (**6**), which were not cytotoxic to the non-transformed cells of the mammary gland, were chosen to study the inhibition of the migration, formation and growth of colonies of the cancer cell lines. Glycoside **2** effectively inhibited the growth of colonies and the migration of the MDA-MB-231 cell line. Compound **6** blocked the growth of colonies of T-47D cells and showed a pronounced antimigration effect on MDA-MB-231 cells. The quantitative structure–activity relationships (QSAR) indicated the strong impact on the activity of the form and size of the molecules, which is connected to the length and architecture of the carbohydrate chain, the distribution of charge on the molecules’ surface and various aspects of hydrogen bond formation, depending on the quantity and positions of the sulfate groups. The QSAR calculations were in good accordance with the observed SAR tendencies.

## 1. Introduction

Sea cucumbers are marine invertebrates (class Holothuroidea, phylum Echinodermata) that produce the characteristic secondary metabolites–triterpene glycosides. The chemical features of these compounds and their various biological activities have attracted the interest of scientists worldwide to triterpene glycosides of sea cucumbers. The number of chemically studied species of holothuroids is steadily increasing, causing the broadening of knowledge on the chemical biodiversity of glycosides [1]. Ongoing investigations of the chemical structures of the glycosides have spurred the development of mass-spectrometry-based metabolomic research on sea cucumbers [2,3,4,5,6] and the creation of mass spectrometry database for sea cucumber triterpene glycosides [7].

Studies of triterpene glycosides, biosynthesized by the sea cucumbers, not only have fundamental scientific interest but also practical applications based on the diverse biological activity of these compounds. Some of them have demonstrated potential antitumor action against different human cancer cells in vitro, initiating apoptosis by diverse signaling pathways, inhibiting the adhesion, migration, invasion and angiogenesis of cancer cells [8,9,10,11,12,13,14,15], or can be used in cancer immunotherapy [16].

The analysis of the biological activity and chemical structures of the glycosides allowed us to reveal the “structure–activity relationships”, demonstrating the impact of different structural parts of the glycosides on the realization of their physiological action [17]. The application of modern computational tools and knowledge of certain chemical features influencing the membranolytic activity of these compounds and their use as substantial descriptors have resulted in the acquisition of QSAR data [18].

Other directions of the research on glycosides from holothuroids have also focused on their biosynthesis [19,20,21,22,23,24] and chemical synthesis [25,26]. All these data indicate the relevance of studying triterpene glycosides from sea cucumbers.

The genus *Psolus* comprises 58 species of sea cucumbers. Four species of the genus have been chemically studied: *P. fabricii* [27,28,29,30], *P. eximius* [31], *P. patagonicus* [32,33] and *P. chitonoides* [34,35,36]. Usually, every new investigation of the glycosidic composition of unstudied species of sea cucumbers results in the finding of dozens of new structural variants of the glycosides. So, the glycosidic composition of another *Psolus* species—*P. peronii*—was studied for the first time, including isolation of the native glycosides and elucidation of their structures. The finding of only one new glycoside peronioside A (**1**) was rather striking, while the rest of the seven isolated compounds had been found previously in *P. fabricii*. Nevertheless, the chemical structure of **1** was elucidated by ^1^H, ^13^C NMR, 1D TOCSY and 2D NMR (^1^H,^1^H-COSY, HMBC, HSQC, ROESY) spectra and the HR-ESI mass spectra analyses. The known psolusosides A (**2**), B (**3**), G (**4**), I (**5**) [28], L (**6**), N (**7**) and P (**8**) [30] were identified through comparison of the obtained NMR data with those published earlier (Figures S9–S15). The cytotoxicity of individual compounds against the human breast cancer cell lines MCF-7, T-47D and triple-negative MDA-MB-231 was tested. Regarding the fact that breast cancer is the most widespread and the leading cause of death from oncologic diseases in women, the search for anti-breast cancer drugs is urgently needed. The inhibition of the migration, formation and growth of colonies of the cancer cells under treatment with the most active compounds (**2** and **6**) from the series was also studied.

## 2. Results and Discussion

### 2.1. Structural Elucidation of the Glycosides

The specimens of *Psolus peronii* were collected in Avacha Bay near Starichkov Island. Sea cucumbers were twice refluxed with EtOH to extract the glycosides (the weight of the dry residue was 355.7 g). The crude glycosidic sum (3.6 g) was obtained after hydrophobic chromatography of the concentrated and degreased extract on a Polychrom-1 column (powdered Teflon, Biolar, Latvia). Its further separation was achieved by using chromatography on Si gel columns (CC), with a stepped gradient of the eluent system CHCl_3_/EtOH/H_2_O at ratios of 100:50:4, 100:75:10, 100:100:17 and 100:125:25 resulting in the obtaining of five subfractions.

**Figure 1:**
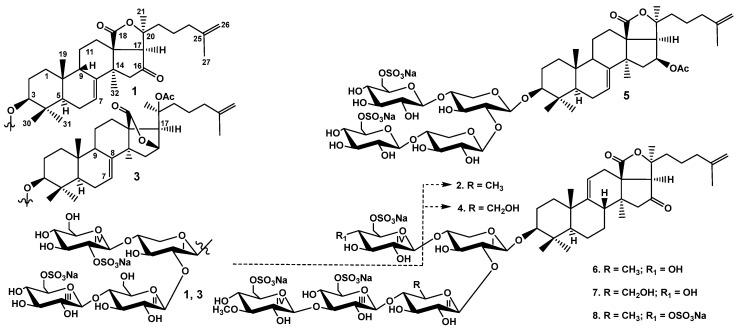
Chemical structures of glycosides isolated from *Psolus peronii:* **1**—peronioside A; **2**—psolusoside A; **3**—psolusoside B; **4**—psolusoside G; **5**—psolusoside I; **6**—psolusoside L; **7**—psolusoside N; **8**—psolusoside P.

Each was repeatedly purified using CC with the solvent system corresponding to a polarity of the subfraction that resulted in the obtaining of fractions 1 (585 mg), 2 (164 mg), 3 (107 mg), 4 (40 mg) and 5 (83 mg). These fractions were subsequently subjected to HPLC on the reversed-phase semipreparative columns Phenomenex Synergi Fusion RP (10 × 250 mm) and Synergi Hydro RP (10 × 250 mm) to give the individual glycosides **1**–**8** (Figure 1).

The molecular formula of peronioside A (**1**) was determined to be C_53_H_80_O_29_S_2_Na_2_ from the [M_2Na_ − Na]^−^ ion peak at *m/z* 1267.4120 (calc. 1267.4130) and the [M_2Na_ − 2Na]^2−^ ion peak at *m/z* 622.2113 (calc. 622.2120) in the (−)HR-ESI-MS (Figure S8). The ^13^C NMR spectrum of **1** indicated the presence of a holostane-type aglycone, i.e., having 18(20)-lactone (signals at δ_C_ 179.3 (C-18) and δ_C_ 83.8 (C-20)), a carbonyl group at C-16 (δ_C_ 214.2 (C-16), 51.9 (C-15), 63.4 (C-17)) and 7(8)- and 25(26)- double bonds (δ_C_ 121.8 (C-7) and 143.9 (C-8), 145.5 (C-25) and 110.4 (C-26)) (Table 1). The HMBC correlations confirmed the positions of the quaternary carbons and functional groups: H-15/C: 13, 14, 16; H-17/C: 12, 16, 18, 20; H-19/C: 10; H-21/C: 20; H-24/C: 25; H-30, H-31/C: 4; H-32/C: 13, 14. The NOE correlations H-3/H-5, H-31; H-9/H-19; H-17/H-21 and H-17/H-32 corroborated configurations of the chiral centers typical of holostane-type aglycones: C: 3, 9, 14, 17, 20. The identical aglycone was found in psolusosides H and J isolated recently from *P. fabricii* [28,30] but discovered for the first time in the glycosides of *Cucumaria japonica* [37].

In the ^1^H and ^13^C NMR spectra of the carbohydrate part of peronioside A (**1**), four anomeric doublets at δ_H_ 4.59–5.14 (*J* = 6.8–8.1 Hz) and the corresponding signals of anomeric carbons at δ_C_ 101.1–104.8 indicated the presence of a tetrasaccharide chain with *β*-configurations of the glycosidic bonds (Table 2). The analysis of the 1D TOCSY spectrum of the first monosaccharide unit attached to C-3 of the aglycone showed it was a xylose residue typical of sea cucumber glycosides. The signals of C-2 Xyl1 and C-4 Xyl1 were deshielded due to the glycosylation effects, demonstrating the branching of the sugar chain of **1** at position 4 of the first monosaccharide residue. Actually, the NOE correlations H-2 Xyl1/H-1 Glc2 and H-4 Xyl1/H-1 Glc4 confirmed the bonding of Xyl1 with the other two monosaccharides. The second, third and fourth monosaccharides in the chain of **1** were the glucose residues that was deduced from the ^1^H-^1^H COSY, ROESY and 1D TOCSY spectra. The positions of the interglycosidic linkages were elucidated by the ROESY and HMBC spectra of **1** (Table 2), where correlations between H-1 of the xylose (Xyl1) and H-3 (C-3) of the aglycone, H-1 of the second monosaccharide residue (glucose, Glc2) and H-2 (C-2) of the xylose (Xyl1), H-1 of the third residue (glucose, Glc3) and H-4 (C-4) of the second residue (glucose, Glc2) and H-1 of the fourth residue (glucose, Glc4) and H-4 (C-4) of the first residue (xylose, Xyl1) were observed, corroborating the presence of the tetrasaccharide branched chain in **1**. The presence of sulfate groups was deduced on the basis of the observed α- and β-shifting effects in the ^13^C NMR spectrum of **1**. The signal of C-6 Glc3 was deshielded to δ_C_ 67.4, while the signal of C-5 Glc3 was shielded to δ_C_ 75.6, which indicated the sulfation of C-6 of this monosaccharide residue. Another sulfate group was attached to C-2 Glc4, causing the deshielding of C-2 Glc4 to δ_C_ 80.6, as well as characteristic shielding of the signal of C-1 Glc4 to δ_C_ 101.1. Actually, comparison of the NMR spectra of the carbohydrate part of **1** with that of psolusoside B [28] showed their coincidence.

The (−)ESI-MS/MS (Figure S8) of **1** showed the fragmentation of the [M_2Na_ − Na]^−^ ion at *m/z* 1267.4 resulted in the appearance of ion peaks at *m/z* 1147.5 [M_2Na_ − Na − NaHSO_4_]^−^, 1003.4 [M_2Na_ − Na − GlcSO_3_Na + H]^−^ and 901.4 [M_2Na_ − Na − GlcSO_3_Na − SO_3_Na + H]^−^, corroborating the presence of two sulfate groups and sulfated glucose as a terminal monosaccharide residue. Further fragmentation of the carbohydrate chain of **1** led to an ion peak at *m/z* 841.4 [M_2Na_ − Na − GlcSO_3_Na − Glc + H]^−^, while ion peaks at *m/z* 799.1 [M_2Na_ − Na − Agl − H]^−^ and 535.1 [M_Na_ − Na − Agl − GlcSO_3_Na]^−^ confirmed the aglycone structure of **1**.

The configurations of the sugars in the glycosides of *P. peronii* were assigned as D on the basis of biogenetic considerations since D configurations of the monosaccharides in psolusoside A (**2**) from *Psolus fabricii* were previously determined [38] and because all the monosaccharide residues in the known sea cucumber triterpene glycosides have D configurations.

All these data indicate that peronioside A (**1**) is 3*β*-*O*-{6-*O*-sodium-sulfate-*β*-D-glucopyranosyl-(1→4)-*β*-D-glucopyranosyl-(1→2)-[2-*O*-sodium-sulfate-*β*-D-glucopyranosyl-(1→4)]-*β*-D-xylopyranosyl}-16-oxoholosta-7,25-diene.

Thus, peronioside A (**1**) is very close structurally and biogenetically to the glycosides of the sea cucumber *P. fabricii*, being a partially desulfated derivative (by C-4 Glc4) of psolusoside J [30]. Moreover, the other seven compounds isolated from the glycosidic fraction of *P. peronii* were found for the first time in the sea cucumber *P. fabricii*. However, the set of glycosides in *P. peronii* was less diverse. Instead of the 32 individual compounds isolated from *P. fabricii*, only 8 substances were obtained from the species under investigation. Nonetheless, the aglycones of the glycosides of *P. peronii* varied by the lactone position (18(20)-lactone in the holostane aglycones of glycosides **1**, **2** and **4**–**8** and 18(16)-lactone in the non-holostane aglycone of psolusoside B (**3**)), by the intranuclear double bond position (7(8)- in peronioside A (**1**), psolusosides B (**3**) and I (**5**) and 9(11)- in the other five compounds) and finally by the substituent at C-16 in the holostane aglycones (a carbonyl group in six glycosides and an *O*-acetic group in psolusoside I (**5**)). The diversity of the oligosaccharide chains of the glycosides of *P. peronii* was provided by their architecture (linear tetrasaccharide chains in psolusosides A (**2**) and G (**4**) and branched tetrasaccharide chains in peronioside A (**1**) and psolusosides B (**3**) and I (**5**)), by the quantity of monosaccharide residues (four monosaccharides in **1**–**5** and five in **6**–**8**), by the sugar composition (different second sugars: quinovose in psolusosides A (**2**), L (**6**) and P (**8**), glucose in peronioside A (**1**), psolusosides B (**3**), G (**4**) and N (**7**)) and xylose in psolusoside I (**5**)) and finally by the quantity of sulfate groups (from two in compounds **1**–**5** and three in glycosides **6** and **7** to four in psolusoside P (**8**)) and their positions. Such chemical diversity of the glycosides indicated the species *P. peronii* possesses powerful and developed enzyme machinery for biosynthesis of the glycosides.

**Figure 2:**
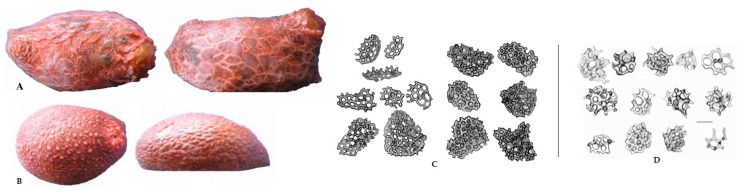
Morphological differences of *P. fabricii* and *P. peronii*. (**A**)—dorsal and lateral view of *P. fabricii*; (**B**)—dorsal and lateral view of *P. peronii*; (**C**)—the ossicles of *P. fabricii*; (**D**)—the ossicles of *P. peronii*. Photos by Stepanov V.G.

The structural closeness of the glycosides of two sea cucumber species—*P. peronii* and *P. fabricii*—pointed out the phylogenetic and systematic similarity of these species. Actually, these two species are closely related but with sufficiently different morphologic features. The body length of *P. fabricii* is up to 100 mm, while *P. peronii*’s length reaches only 60 mm. *P. fabricii* has a higher mouth extremity and larger body scales than *P. peronii*. The small scales of *P. peronii* are covered with fine granules and tightened with thin “skin”. The ossicle shapes of these species are also different (Figure 2). So, the phenotypic distinctions, as well as the availability of the new glycoside **1** in *P. peronii*, corroborated the separateness of these species.

### 2.2. Bioactivity of the Glycosides

The cytotoxic activity of glycosides **1**–**8** and the partially desulfated psolusoside B (DS-psolusoside B) was tested against human erythrocytes and human breast cancer cell lines (MCF-7, T-47D and triple-negative MDA-MB-231), as well as against the non-transformed epithelial cells of the mammary gland MCF-10A (Table 3). Cisplatin was used as the positive control in the testing of cytotoxic activity against the cancer cells and MCF-10A cells. The known psolusosides **2**–**8** can be considered positive controls in the testing of hemolytic activity since their hemolytic activity has previously been examined [28,30]. The activity of the glycosides against all the cell lines was examined by MTT assay.

Peronioside A (**1**) demonstrated moderate hemolytic activity (Table 3) due to the presence of branched at C-4 Xyl1 carbohydrate chain and the sulfate group at C-2 Glc4, features decreasing its bioactivity, as was noted earlier [17,28]. Psolusoside B (**3**) was almost not active because of the presence of a non-holostane aglycone in addition to the same sugar chain as in **1**. The aglycone’s negative impact on the membranotropic activity was not compensated for even by the deletion of the sulfate group at C-2 Glc4 in the partially desulfated psolusoside B (assigned the ^13^C NMR spectrum—Figure S16), obtained after its spontaneous formation in the process of desalting of psolusoside B (**3**) after HPLC using hydrophobic chromatography on Polychrom-1 (see the Materials and Methods section). Psolusoside I (**5**), having the same architecture of oligosaccharide moiety as **1** and **3** but lacking a sulfate group at C-2 Glc4 and having it instead at C-6 Glc4, as well as the xylose as the second unit in the chain and the holostane aglycone, was significantly more active than **1** and **3**. This demonstrates the positive impact of the holostane aglycone and the influence of different positions of the sulfate group. The most active in relation to the erythrocytes, psolusosides A (**2**), G (**4**) and L (**6**), have identical holostane aglycones. The linear tetrasaccharide chains of **2** and **4** differed by the second monosaccharide residue: quinovose in **2** made it twice more active, than **4**, having a glucose. The combination of linear sugar chains and holostane aglycones in these compounds provided their high hemolytic activity. Similar structure–activity relationships (SAR) were observed for the pentaosides—psolusosides L (**6**), N (**7**) and P (**8**). The same structural feature in pair **6** and **7** as pair **2** and **4** (quinovose or glucose as the second unit) caused a more than nine-fold difference in the activities of **6** and **7**, which can be explained by the absence of the positive impact of the linear tetrasaccharide chain, as was observed for glycosides **2** and **4**. The negative impact of the fourth sulfate group attached to C-4 Glc5, in addition to another sulfate group at C-6 Glc5, in psolusoside P (**8**) caused a four-fold decrease in its hemolytic activity in comparison with that of psolusoside L (**6**), having only three sulfate groups, in the same positions as in **8**.

Psolusosides A (**2**) and L (**6**) were the most active compounds against cancer cells from all the tested lines. Of note, compound **2** was not cytotoxic and compound **6** was slightly cytotoxic to the non-transformed cells of the mammary gland MCF-10A (Table 3). Psolusoside A (**2**) demonstrated a stronger cytotoxicity against triple-negative breast cancer (MDA-MD-231 line), with the highest selectivity index, 8.21, at 24 h of exposition (Table 4). Meanwhile, psolusoside L (**6**) was almost equally active against T-47D and MDA-MB-231 (selectivity indexes of 6.07 and 5.18, correspondingly).

**Figure 3:**
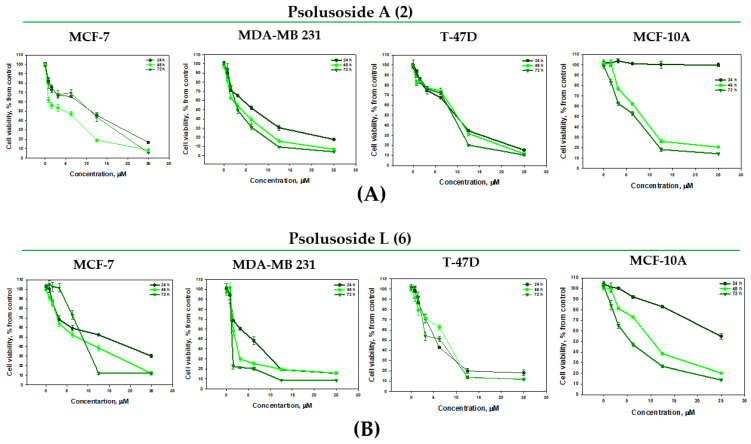
Cytotoxic effect of glycosides against breast cancer cells: (**A**) psolusoside A (**2**) against MCF-7, MDA-MB-231, T-47D, MCF-10A cells; (**B**) psolusoside L (**6**) against MCF-7, MDA-MB-231, T-47D, MCF-10A cells for 24 h, 48 h and 72 h. All experiments were carried out in triplicate. The data are presented as means ± SEM.

The antiproliferative properties of **2** and **6** were studied as a result of the prolonged incubation of the cells for 48 and 72 h with the glycosides (Figure 3). They did not lose their cytotoxicity over time but inhibited the proliferation of the MCF-10A cells. The antiproliferative effects of psolusosides A (**2**) and L (**6**) against the MDA-MB-231 cells for 48 and 72 h were observed as approximately two-fold and five-fold increases in their IC_50_ (3.87 ± 0.38 and 3.27 ± 0.27 μM and 1.67 ± 0.16 and 1.24 ± 0.02 μM), correspondingly (Figure 3). Glycoside **6** inhibited the proliferation of the MCF-7 cells as well (Figure 3B).

**Figure 4:**
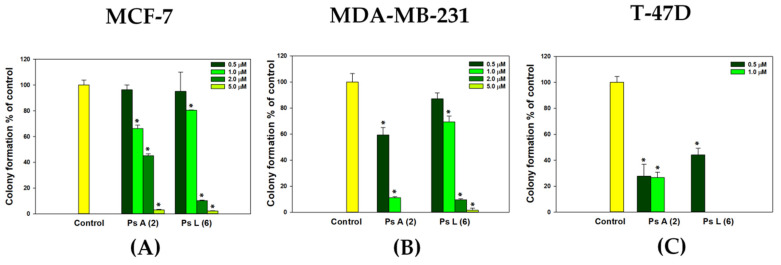
The number of MCF-7 (**A**), MDA-MB-231 (**B**) and T-47D (C) cell colonies under the treatment with different concentrations of psolusosides A (**2**) and L (**6**). Data are presented as means ± SEM. * *p* value ≤ 0.05 considered significant.

To observe the long-term effects of psolusosides A (**2**) and L (**6**) on the breast cancer cell lines, a colony formation assay was performed, illustrating the ability of the tumor cells to grow uncontrollably and the influence of the tested glycosides on this process. The cells were treated with concentrations of glycosides **2** and **6** below their IC_50_ values and exposed for 14 days. Concentrations of 2 and 5 µM of the tested compounds completely inhibited the formation of colonies of all the cell lines investigated (Figure 4). A dose-dependent effect was observed for both compounds in relation to the MCF-7 cells (Figure 4A) (inhibition of colony formation by 33.76 ± 2.64% and 19.59 ± 0.24% at a concentration of 1 μM, by 54.88 ± 1.92% and 89.67 ± 0.28 at a concentration of 2 μM and by 96.88 ± 0.96% and 97.83 ± 0.25% at a concentration of 5 μM of psolusosides A (**2**) and L (**6**), respectively). Glycoside **2** was more effective against the MDA-MB-231 cells than **6** (Figure 4B), inhibiting the growth of colonies by 40.56 ± 5.62% at a concentration of 0.5 μM and by 88.76 ± 0.81% at a concentration of 1 μM. Psolusoside L (**6**) blocked the growth of MDA-MB-231 cell colonies by 90.36 ± 0.92% at a higher concentration of 2 μM. The greatest effect was observed for psolusoside L (**6**) against the T-47D cell line: the glycoside completely blocked the growth of colonies even at concentration of 1 μM (Figure 4C and Figure S17). Thus, the glycosides under investigation showed high effectiveness in the inhibition of colony formation, related to the ability of cancer cells to grow uncontrollably and divide in living organisms.

**Figure 5:**
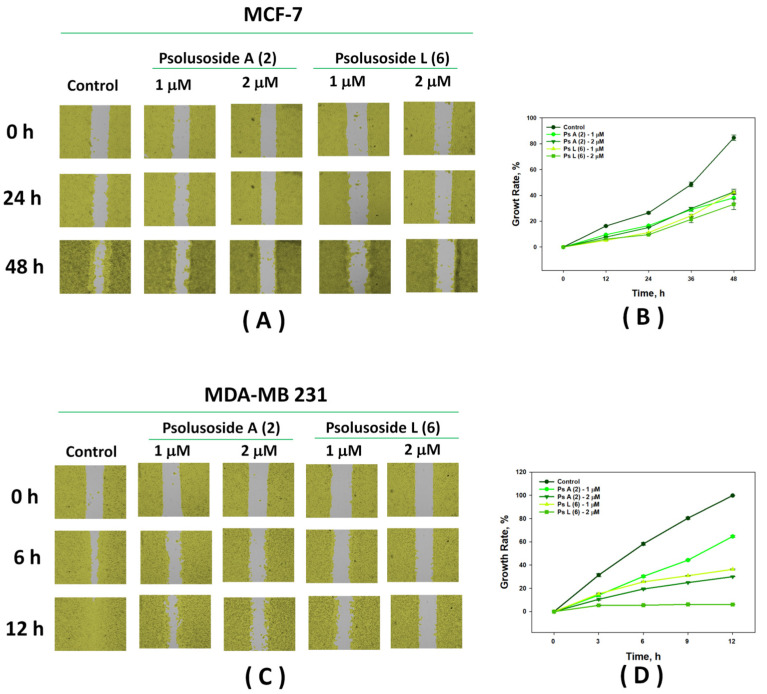
Migration of MCF-7 (**A**,**B**) and MDA-MB-231 (**C**,**D**) cells into wound areas observed with real-time live cell imaging system Juli Stage: (**A**) 0, 24, and 48 h for MCF-7 cells after treatment with concentrations of 1 and 2 μM of psolusosides A (**2**) and L (**6**). (**B**) Time-course curves of MCF-7 cell migration into the wound area. (**C**) 0, 6 and 12 h for MDA-MB-231 cells after treatment with concentrations of 1 and 2 μM of psolusosides A (**2**) and L (**6**). (**D**) Time-course curves of MDA-MB-231 cell migration into the wound area. All experiments were carried out in triplicate. The data are presented as means ± SEM.

The scratch method is used to study the action of compounds with potential antitumor activity on cell motility and cell–cell interactions, which are connected to the ability of cancer cells to migrate. The search for substances capable of inhibiting their migration is relevant to the prevention of metastasis. Wound healing analysis involved comparison of the areas of migrating cells in the control and under treatment with the tested compounds at different time intervals. Psolusosides A (**2**) and L (**6**) were found to be effective in reducing the migration of the MDA-MB-231 and MCF-7 cells at non-cytotoxic concentrations (Figure 5). Thus, in the control, the MCF-7 cells migrated by 84.60 ± 2.12% within 48 h. Both glycosides, **2** and **6**, reduced the migration of these cells to the scratch area by 33–42% at concentrations of 1 and 2 μM within 48 h (Figure 5A,B). 

The MDA-MB-231 cells migrated to the wound area much faster, demostrating an overgrowth of the starting point by 58.26 ± 1.11% after 6 h and by 99.83 ± 0.08% after 12 h in the control group. The addition of glycosides **2** and **6** to the cells significantly inhibited migration. Psolusoside A (**2**) reduced the overgrowth to 30.01 ± 0.16% at a concentration of 2 μM within 12 h. Psolusoside L (**6**) showed a more pronounced effect and almost completely blocked the movement of the MDA-MB-231 triple-negative breast cancer cells (6.03 ± 0.02% of overgrowth) at the same concentration and in the same time (Figure 5C,D, Videos S1–S5).

So, psolusosides A (**2**) and L (**6**) showed promising anti-breast cancer activity, strongly inhibiting the growth of colonies of the T-47D and MDA-MB-231 cell lines and significantly slowing down the migration of the MCF-7 and MDA-MB-231 cell lines.

### 2.3. Quantitative Structure–Activity Relationships

**Figure 6:**
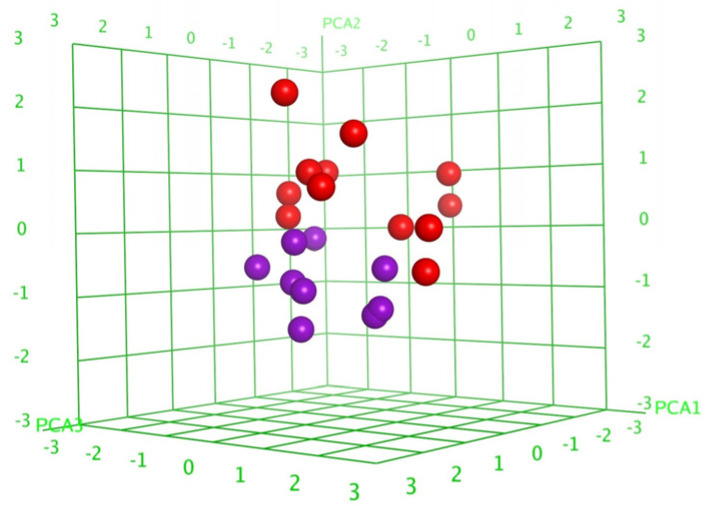
Three-dimensional plot of hemolytic activity (pIC_50_) dependence on the principal component values (PCA1—PCA3) calculated for 21 conformational forms of nine glycosides (**1**–**8** and DS-psolusoside B). The glycosides demonstrating hemolytic activity with IC_50_ ≤ 10 µM were outlined as active and are marked in red, while the rest are marked in violet.

To compare the trends in the observed SAR, the quantitative relationships (QSAR) were calculated for the series of compounds **1**–**8,** as well as DS-psolusoside B, on the basis of correlational analysis and multivariate regression analysis methods using molecular 2D and 3D descriptors (379 in total), describing the atomic, spatial (conformational) and physicochemical properties of the compounds. The principal component analysis (PCA, Figure 6) reduced the number of descriptors to 109, including only those making a significant contribution to the membranolytic properties of the glycosides, and resulted in the division of the tested compounds into two groups, that confirmed the correct descriptor choice. A linear QSAR model was built with the QuaSAR-Model tool and the PLS, PCR and Binary algorithms of the MOE 2020.0901 CCG software [39] using the selected descriptors. The best, the PLS model, fitted well with the experimental data on the hemolytic activities of the tested glycosides and was characterized by good statistical parameters (correlation coefficient r^2^ = 0.9997 and RMSE = 0.0099, cross-validation coefficient r^2^_cros_ = 0.8443 and RMSE_cros_ = 0.2400) (Figure S18). The QSAR model comprised 87 descriptors, with 66 of those having the biggest contribution. A further reduction in the number of descriptors resulted in deterioration of the quality of the correlation model (up to r^2^ = 0.6187 and RMSE = 0.42575), indicating the considerable impact of multiple negligible effects of plenty of descriptors on the total result. 

The correlational analysis revealed the direct positive correlation between the hemolytic activities of the tested compounds in vitro and such descriptors as the molecular refractivity, the octanol/water partition coefficient (logP) [40], the partial charge distribution on the van der Waals (VDW) surface area (PEOE group), hydrophilic volume (vsurf_W, Å^3^), interaction field volume, polar surface area (TPSA, Å^2^) and weinerPath (the sum of the lengths of the shortest paths between all pairs of heavy atoms). In contrast, the diameter of the molecule, the principal moment of inertia (pmi), describing the different aspects of the molecular shape, the VDW surface area (Å^2^), the molecular VDW volume (Å^3^), the lowest hydrophobic energy, the approximation of the sum of the VDW surface areas of hydrophobic atoms (PEOE_VSA_FHYD, Å^2^) and polar volume were found to negatively correlate with the activity. 

As was mentioned above, the structure of the carbohydrate chain substantially affects the membranolytic activity of the glycosides. The constructed QSAR model indicated one of the fundamentally important groups of descriptors was related to the molecular size and shape: diameter, surface rugosity (surf_R [41]) and the moment of inertia of the molecule (pmi). Actually, these parameters are directly related to the length of the linear part and the availability of the branching of carbohydrate chains. The presence of a tetrasaccharide linear sugar moiety showed a strong positive correlation with the activity of the glycosides. Meanwhile, the appearance of a branch point at C-4 of the first xylose residue led to a dramatic change in the shape of the molecule and negatively affected its hemolytic activity. 

The impact of sulfate groups on the membranolytic properties of the glycosides was ambiguous. The total number of sulfate groups showed only a weak positive correlation with the activity of the tested compounds. The presence of a sulfate group at C-6 of the terminal 3-*O*-methylglucose residue correlated strongly positively, whereas the sulfation of the C-4 or C-6 positions of the glucose unit (Glc4 or Glc5) bonded to C-4 Xyl1 did not demonstrate a significant correlation, in opposition to the sulfation of this monosaccharide by the C-2 position, which was correlated strongly negatively. Since a negative correlation between the contribution of electrostatic interactions and angular bending to the potential energy of the molecule was observed, this most likely indicated the sulfate group at C-2 of the terminal glucose residue in the upper semi-chain caused the electrostatic repulsion of the sulfate groups attached to the third and fourth residues in the bottom semi-chain, which led to the “expansion” of the molecule, resulting in a decrease in hemolytic activity, as was observed in the case of peronioside A (**1**) and psolusoside B (**3**).

**Figure 7:**
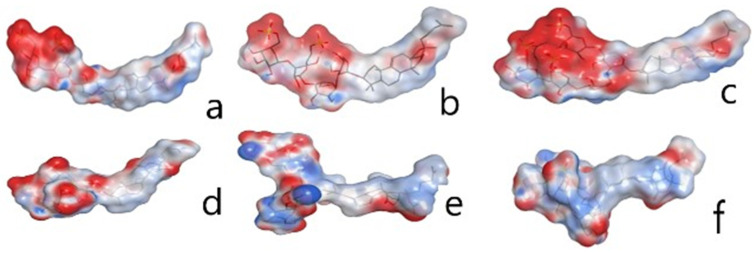
The charge distribution on the molecular surface of glycosides from sea cucumber *P. peronii*. Molecular surfaces are presented as a contour and colored according to the charge distribution (positive is blue, negative is red). Molecular structures are presented as sticks: (**a**)—psolusoside A (**2**); (**b**)—psolusoside G (**4**); (**c**)—psolusoside L (**6**); (**d**)—peronioside A (**1**); (**e**)—psolusoside B (**3**); (**f**)—psolusoside N (**7**).

Noticeably, the QSAR model includes the following descriptors—CACA, vsurf_HB, E_strain, VDW, PEOE and dipole—reflecting the contribution of hydrogen bonds (HBs) and steric and Coulomb interactions. The most active compounds, **2**, **4** and **6,** are characterized by highly grouped negatively charged patches on their surfaces, whereas glycosides **1**, **3** and **7** have locally distributed areas of positive and negative charge along their molecular surface (Figure 7), which are a plausible cause of their decreased activity.

The complex role of hydrogen bond (HB) donors and acceptors should also be noted. On the one hand, HB donors (capable of interactions with the other surrounding molecules) have been shown to make a positive contribution to the predicted activity, as opposed to the contribution of strong intramolecular HBs (vsurf_D1 parameter). Thus, the DS-psolusoside B carbohydrate chain is capable of forming an extensive network of intramolecular HBs, unlike more active glycosides (Figure S19).

The QSAR also confirmed the strong influence of the second monosaccacharide residue (quinovose, glucose or xylose) on the activity, which was previously observed [17]. Hence, psolusoside A (**2**), having quinovose as the second sugar unit, was more active than psolusoside G (**4**), with glucose in this position. Noticeably, according to the correlational analysis, this effect was more considerable compared to the negative impact of carbohydrate chain branching. So, the presence of quinovose can be considered a special “activity protection” property, as illustrated by psolusosides L (**6**) and P (**8**), with a second quinovose residue, which are more active than psolusoside N (7), with a second glucose unit.

It is well known that the ability of sea cucumber glycosides to destroy erythrocyte membranes is largely dependent on the structural features of its aglycones [17]. To clarify this impact, additional descriptors reflecting the lactone and intranuclear double bond positions, as well as the presence of functional groups, were introduced alongside those included in the MOE 2020.0901 package [39]. The descriptors attributed to the presence of 18(20)-lactone and a 16-oxo group turned out to be the most significant positive variables for the predicted hemolytic activity of the glycosides in the QSAR model. The introduction of 18(16)-lactone resulted in a decrease in the activity according to both the observed and calculated SAR, obviously due to the rigidity of such aglycones, which hinders the adaptation of the molecule to the membrane environment.

## 3. Materials and Methods

### 3.1. General Experimental Procedures

Specific rotation, Perkin-Elmer 343 Polarimeter; NMR, Avance III 700 Bruker FT-NMR (Bruker BioSpin GmbH, Rheinstetten, Germany) (700.13/176.04 MHz) (^1^H/^13^C) spectrometers were used with tetramethylsilane as the internal standard; ESI MS (positive and negative ion modes), the Agilent 6510 Q-TOF apparatus was used with a sample concentration of 0.01 mg/mL; HPLC, Agilent 1260 Infinity II equipped with a differential refractometer (Agilent Technology, Santa Clara, CA, USA); columns, Phenomenex Synergi Fusion RP (10 *×* 250 mm, 5 µm), Phenomenex Synergi Hydro RP (10 *×* 250 mm, 5 µm) (Phenomenex, Torrance, CA, USA) and Supelco Discovery HS F5-5 (10 *×* 250 mm, 5 µm) (Supelco, Bellefonte, PA, USA).

### 3.2. Animals and Cells

Specimens of the sea cucumber *Psolus peronii* (family Psolidae; order Dendrochirotida) were collected in Avacha Bay near Starichkov Island. Sampling was performed by scuba diving in June 2014 at a depth of 10–15 m. Sea cucumbers were collected and taxonomically identified by V.G. Stepanov. Voucher specimens are preserved in the Pacific Institute of Geography, Kamchatka Branch, Petropavlovsk-Kamchatsky, Russia.

Human erythrocytes were purchased from the Station of Blood Transfusion in Vladivostok. The human breast cancer cell lines T-47D HTB-133^TM^, MCF-7 HTB-22^TM^ and MDA-MB-231 CRM-HTB-26^TM^ and the mammary epithelial cell line MCF-10A CRL-10317 were received from ATCC (Manassas, VA, USA). The T-47D cell line was cultured in RPMI medium with 1% penicillin/streptomycin (Biolot, St. Petersburg, Russia) and 10% fetal bovine serum (FBS) (Biolot, St. Petersburg, Russia). Cells from the MCF-7 and MDA-MB-231 lines were cultured in MEM (Minimum Essential Medium) with 1% penicillin/streptomycin sulfate (Biolot, St. Petersburg, Russia) and with fetal bovine serum (Biolot, St. Petersburg, Russia) to a final concentration of 10%. The MCF-10A cells were cultured in DMEM/F12 medium with 10% FBS, 20 ng/mL EGF, 0.5 mg/mL hydrocortisone, 100 ng/mL cholera toxin, 10 μg/mL insulin and 1% penicillin/streptomycin (Bioinnlabs, Russia).

### 3.3. Extraction and Isolation

The sea cucumbers were frozen at −20 °C after collection and then minced and extracted twice with refluxing 60% EtOH (the animal’s dry weight = 355.7 g). The extracts were combined and evaporated to oily residuum, which was subjected to Si gel column chromatography (CC) to remove the lipids and sterol sulfates with CHCl_3_/EtOH (9/1). The glycosides were eluted from the column with CHCl_3_/EtOH/H_2_O (100:150:40). The obtained sum was chromatographed on a Polychrom-1 column (powdered Teflon, Biolar, Latvia) to remove salts and polar impurities with water elution. The elution of the glycosides was achieved with 50% EtOH. The weight of the obtained glycosidic sum was 3600 mg. Its further separation was achieved by using chromatography on Si gel columns (CC), with a stepped gradient of the eluent system CHCl_3_/EtOH/H_2_O at ratios of 100:50:4, 100:75:10, 100:100:17 and 100:125:25 resulting in the obtaining of five subfractions. Each of them was repeatedly purified by CC with a solvent system corresponding to a polarity of the subfraction that resulted in the acquisition of fractions 1 (585 mg), 2 (164 mg), 3 (107 mg), 4 (40 mg) and 5 (83 mg). Subsequent HPLC of fraction 1 on the Phenomenex Synergi Fusion RP column (10 × 205 mm) with CH_3_CN/H_2_O/NH_4_OAc (1 M water solution) (38/60/2) as the mobile phase gave psolusoside A (**2**) (12.7 mg, Rt 20.5 min) and three other subfractions (1.2–1.4). HPLC of subfraction 1.4 on the same column with the same solvents as the mobile phase but at a ratio of 39/58/3 resulted in the isolation of 2.1 mg of psolusoside G (**4**) (Rt 15.0 min). HPLC of subfractions 1.3 and 1.2 with a ratio of 37/60/3 of the same solvents in the mobile phase allowed us to obtain 2.0 mg of psolusoside I (**5**) (Rt 14.8 min). HPLC of fraction 2 on the same column with CH_3_CN/H_2_O/NH_4_OAc (1 M water solution) (38/59/3) as the mobile phase resulted in the obtaining of subfractions 2.1 and 2.2. Subsequent chromatography of subfractions 2.2 and 2.1 on the Phenomenex Synergi Hydro RP column (10 × 205 mm) with the same mobile phase gave peronioside A (**1**) (2.3 mg, Rt 13.3 min) and psolusoside B (18.3 mg, Rt 12.5 min), correspondingly. Fraction 3 gave 2.8 mg of psolusoside L (**6**) (Rt 18.0 min) after its separation by HPLC on the Phenomenex Synergi Fusion RP column (10 × 205 mm) with CH_3_CN/H_2_O/NH_4_OAc (1 M water solution) (38/59/3) as the mobile phase. For HPLC of fraction 4 on the same column, a mobile phase consisting of CH_3_CN/H_2_O/NH_4_OAc (1 M water solution) at a ratio of 33/54/3 was used to give 5.1 mg of psolusoside N (**7**) (Rt 16.4 min). Psolusoside P (**8**) (3.7 mg, Rt 14.0 min) was obtained from the most polar fraction 5 as result of its separation on the Phenomenex Synergi Fusion RP column with CH_3_CN/H_2_O/NH_4_OAc (1 M water solution) (34/62/4) as the mobile phase, followed by HPLC on the Supelco Discovery HS F5-5 column with the same solvents at a ratio of 29/68/3 as the mobile phase.

Each obtained individual glycoside was subsequently desalted from the ammonium acetate before mass spectra registration using chromatography on the Polychrom-1 column. Additionally, the compounds were stabilized after loading them onto the Polychrom-1 column by washing with 10% NaHCO_3_ solution for the prevention of spontaneous desulfation. This occured with psolusoside B (**3**) resulted in DS-psolusoside B formation (the NMR data of the desulfated derivative were obtained (Figure S16)).

#### Peronioside A (**1**)

Colorless powder: [α]_D_^20^ −20 (*c* 0.1, 50% MeOH). NMR: Table 1 and Table 2. (−)HR-ESI-MS *m/z*: 1267.4120 (calc. 1267.4130), [M_2Na_ − Na]^−^, 622.2113 (calc. 622.2120) [M_2Na_−2Na]^2−^; (−)ESI-MS/MS *m/z*: 1147.5 [M_2Na_ − Na − NaHSO_4_]^−^, 1003.4 [M_2Na_ − Na − C_6_H_10_O_8_SNa + H]^−^, 901.4 [M_2Na_ − Na − C_6_H_10_O_8_Sna − SO_3_Na + H]^−^, 841.4 [M_2Na_ − Na − C_6_H_10_O_8_Sna − C_6_H_10_O_5_ + H]^−^, 799.1 [M_2Na_ − Na − C_30_H_43_O_4_ − H]^−^, 535.1 [M_Na_ − Na − C_30_H_43_O_4_ − C_6_H_10_O_8_SNa]^−^.

### 3.4. Hemolytic Activity

A total of 450 g of human blood (B(III) Rh+) was used to obtain erythrocytes by centrifuging it three times for 5 min with phosphate-buffered saline (PBS) (pH 7.4) at 4 °C on the centrifuge LABOFUGE 400R (Heraeus, Hanau, Germany). Ice-cold PBS (pH 7.4) was used for resuspension of the erythrocyte residue to a final optical density of 1.5 at 700 nm, which was kept on ice. Then, 20 µL of the tested compound solutions was added to 180 µL of the erythrocyte suspension in V-bottom 96-well plates and exposed for 1 h at 37 °C. The layers were separated by centrifugation at 900 g for 10 min on the laboratory centrifuge LMC-3000 (Biosan, Riga, Latvia). A total of 100 µL of the supernatant was carefully decanted and transferred into a new flat plate. The values of erythrocyte lysis were measured on a microplate photometer Multiskan FC (Thermo Fisher Scientific, Waltham, MA, USA) at λ = 570 nm as the hemoglobin concentration in the supernatant. The effective dose, causing lysis of 50% of the erythrocytes (IC_50_), was calculated with SigmaPlot 14.0 software. All the experiments were carried out in triple repetitions; *p* ≤ 0.05.

### 3.5. Cytotoxic Activity (MTT Assay)

Solutions (20 μL) of the tested substances at different concentrations and the cell suspension (200 μL) were added to the wells of 96-well plates and incubated overnight at 37 °C and 5% CO_2_. After incubation, the cells were precipitated by centrifugation, 200 μL of medium from each well was collected and 100 μL of pure medium was added. Then, 10 μL of MTT (3-(4,5-dimethylthiazol-2-yl)- 2,5-diphenyltetrazolium bromide) solution at 5 μg/mL (Sigma, St. Louis, MO, USA) was added into each well. The plate was incubated for 4 h; after that, 100 μL of SDS-HCl was added to each well, and the plate was incubated at 37 °C for 4–18 h. The optical density was measured at 570 nm and 630–690 nm. The cytotoxic activity of the substances was calculated as the concentration that caused 50% metabolic cell activity inhibition (IC_50_).

### 3.6. Colonogenic Assay

The cancer cells (MDA-MB-231, MCF-7 and T-47D lines) were cultured on 6-well plates at a density of 1 × 10^3^ per well in control media (MEM media, 10% FBS, 10,000 U/mL of penicillin and 10,000 mg/mL of streptomycin) or in media supplemented with different concentrations of the glycosides. The cells were incubated for 14 days at 37 °C in a 5% CO_2_ humidified incubator. Then, the colonies were fixed with methanol (25 min), stained with a 0.5% solution of crystal violet (25 min), washed with H_2_O and dried in air. Counting of the grown colonies was carried out using a BIO-PRINT-Cx4 Edge-Fixed Pad-Container (Vilber, Collegien, France) device and using the software Bio-Vision SOFTWARE User and service manual-v18.01 (Vilber, Collegien, France). The results are presented as % of colony inhibition in relation to the control.

### 3.7. Wound Healing Assay

To analyze the influence of the tested compounds on tumor cell migration, MCF-7 and MDA-MB-231 (5 × 10^5^) cells attached to the plate’s plastic bottom were separated using a silicone insert from special migration plates (Culture-insert 2Well 24, ibiTreat). When the insert was removed, a gap of 500 ± 50 mm (according to the manufacturer’s data) between the cells was left. After 24 h, the cells were washed twice with PBS to remove cell debris and floating cells, treated with 1 and 2 μM of psolusosides A (**2**) and L (**6**) and placed in the real-time live cell imaging system Juli Stage (NanoEntek, Seoul, South Korea). Cells treated with the culture medium only were used as the control. The cell migration was analyzed in real time (15 min for each cycle). For the MDA-MB-231 cells, observations were carried out for 12 h (until complete healing of the wound area in the control); for MCF-7 cells, this time was 48 h. The results were processed using the program Juli^TM^ Stat (NanoEntek, South Korea).

### 3.8. Building a QSAR Model

The QuaSAR-Descriptor and MOE-QuaSAR-Model tools of the MOE 2020.0901 package (Chemical Computing Group, Montreal, Canada) [39] were applied to set up the QSAR model for the set of 9 glycosides. The procedure involved the following steps: charge calculation and structure optimization, glycoside conformational search and optimization, descriptor calculation, correlational analysis, principal component analysis (PCA), removing descriptors collinear with another descriptor (unnecessary descriptors), building the QSAR model and model cross-validation, removing descriptors not contributing to the model and model checking by making a graph showing the correlation between the model-predicted value and the experimental activity value, expressed as pIC_50_. For optimization of the conformations of the glycosides, the LowModeMD Search method [42] was used, which generates conformations using a short ~1 ps run of Molecular Dynamics (MD) at a constant temperature, followed by an all-atom energy minimization with a cut-off of 0.01 kcal. Spatial structures sharing minimal energy were considered the most probable.

## 4. Conclusions

Eight individual compounds **1**–**8**—di-, tri- and tetrasulfated glycosides—were isolated from the sea cucumber *Psolus peronii*. One of the compounds, peronioside A (**1**), is a new combination of a known sugar moiety (found first in psolusoside B (**3**)) and a known aglycone (found earlier in psolusosides H and J) and is a desulfated derivative of psolusoside J. The other compounds **2**–**8** were identical to some of the glycosides found in *P. fabricii* [28,30], indicating the closeness of the sea cucumber species *P. peronii* and *P. fabricii*.

The glycosides of *P. peronii* were chemically diverse, including four aglycone types and seven carbohydrate chains, indicating the organism as producer possesses developed enzyme machinery for biosynthesis of the glycosides. The presence of aglycones with both types of intranuclear double bonds (7(8)- and 9(11)-) indicated that two oxidosqualencyclases are active. So, if necessary, the diversity of glycosides of *P. peronii* can be easily increased. It is known that glycosides perform adaptive, regulative and defensive functions in sea cucumbers [24]. Therefore, the glycosidic composition of the organisms depends on seasonal and ecological factors, with these changing causing modifications in the biosynthesis of the glycosides. When sea cucumbers are fixed for glycoside extraction, we can obtain the molecular fingerprint of the glycosidic composition inherent to a certain group of individuals of species living in certain conditions. 

Psolusosides A (**2**) and L (**6**) showed promising anti-breast cancer activity, strongly inhibiting the growth of colonies of the T-47D and MDA-MB-231 cell lines and significantly slowing down the migration of the MCF-7 and MDA-MB-231 cell lines.

The structure–activity relationships (SAR) analyzed for the series of tested compounds corroborated the positive impact of holostane-type aglycones and linear carbohydrate chains and demonstrated the different influences on the membranolytic activity of the sulfate group positions and monosaccharide composition. The QSAR calculations, taking into consideration the wide range of physicochemical properties of the glycosidic molecules, were in good accordance with the observed SAR tendencies. The QSAR data indicated the effect of a huge number of physico-chemical parameters determined by the chemical structures of the compounds, with different impacts on the biological activity, suggesting the complex nature of the structure–activity relationships.

## Figures and Tables

**Table 1:** ^13^C and ^1^H NMR chemical shifts, HMBC and ROESY correlations of aglycone moiety of peronioside A (**1**).

Position	δ_C_ mult. ^a^	δ_H_ mult. (*J* in Hz) ^b^	HMBC	ROESY
1	35.7 CH_2_	1.36 m		H-19
		1.32 m		
2	26.8 CH_2_	2.01 m		
		1.86 m		H-19, H-30
3	89.3 CH	3.15 dd (3.9; 11.7)	C: 31, C:1 Xyl1	H-1, H-5, H-31, H1-Xyl1
4	39.3 C			
5	48.2 CH	0.90 brd (11.5)		H-1, H-3, H-31
6	23.1 CH_2_	1.88 m		H-31
7	121.8 CH	5.92 m		
8	143.9 C			
9	47.0 CH	3.52 brd (14.1)		H-6, H-12, H-19
10	35.5 C			
11	22.3 CH_2_	1.83 m		H-1
12	29.5 CH_2_	2.22 m	C: 18	H-17, H-21, H-32
		1.57 m		H-32
13	56.8 C			
14	45.6 C			
15	51.9 CH_2_	2.66 d (15.4)	C: 13, 16, 17, 32	H-7, H-32
		2.31 d (15.4)	C: 14, 16, 32	
16	214.2 C			
17	63.4 CH	2.90 s	C: 12, 13, 16, 18, 20, 21	H-12, H-21, H-22, H-32
18	179.3 C			
19	23.9 CH_3_	1.08 s	C: 1, 5, 9, 10	H-2, H-6, H-9
20	83.8 C			
21	26.1 CH_3_	1.48 s	C: 17, 20, 22	H-12, H-17
22	38.2 CH_2_	1.69 m		H-17, H-24
		1.54 m		
23	22.1 CH_2_	1.71 m		
		1.42 m		
24	37.8 CH_2_	1.88 m	C: 23, 25, 26, 27	H-22
25	145.5 C			
26	110.4 CH_2_	4.69 brs		
		4.68 brs		
27	22.1 CH_3_	1.62 s	C: 24, 25, 26	
30	17.2 CH_3_	1.03 s	C: 3, 4, 5, 31	H-2, H-31
31	28.5 CH_3_	1.14 s	C: 3, 4, 5, 30	H-3, H-5, H-6, H-30, H-1 Xyl1
32	31.8 CH_3_	1.17 s	C: 8, 13, 14, 15	H-7, H-12, H-15, H-17

^a^ Recorded at 176.04 MHz in C_5_D_5_N/D_2_O (4/1). ^b^ Recorded at 700.13 MHz in C_5_D_5_N/D_2_O (4/1). The original spectra of **1** are provided in Figures S1–S7.

**Table 2:** ^13^C and ^1^H NMR chemical shifts, HMBC and ROESY correlations of carbohydrate moiety of peronioside A (**1**).

Atom	δ_C_ mult. ^a, b, c^	δ_H_ mult. (*J* in Hz) ^d^	HMBC	ROESY
Xyl1 (1→C-3)				
1	104.8 CH	4.59 d (7.7)	C: 3	H-3; H-3, 5 Xyl1
2	**81.1** CH	4.05 t (8.8)	C: 1 Glc2; 1,3 Xyl1	H-1 Glc2
3	75.2 CH	4.23 t (8.8)	C: 2, 4 Xyl1	H-1, 5 Xyl1
4	**78.7** CH	4.12 m	C: 1 Glc4	H-1 Glc4
5	63.8 CH_2_	4.49 m		
		3.78 dd (9.9; 12.7)		H-1, 3 Xyl1
Glc2 (1→2Xyl1)				
1	104.2 CH	5.14 d (8.1)	C: 2 Xyl1	H-2 Xyl1; H-3, 5 Glc2
2	75.3 CH	3.86 t (8.1)	C: 1, 3 Glc2	
3	75.3 CH	3.99 t (8.1)	C: 2, 4 Glc2	H-1, 5 Glc2
4	**82.2** CH	3.90 t (8.1)	C: 3 Glc2; 1 Glc3	H-1 Glc3; H-6 Glc2
5	75.9 CH	3.72 m		H-1, 3 Glc2
6	61.5 CH_2_	4.33 d (10.2)		
		4.28 m		
Glc3 (1→4Glc2)				
1	104.6 CH	4.84 d (6.8)	C: 4 Glc2	H-4 Glc2; H-3, 5 Glc3
2	74.2 CH	3.81 t (9.7)	C: 1, 3 Glc3	H-4 Glc3
3	76.8 CH	4.10 t (9.7)	C: 2, 4 Glc3	H-1 Glc3
4	70.8 CH	3.94 t (9.7)	C: 3, 5, 6 Glc3	H-6 Glc3
5	75.6 CH	4.04 m		H-1 Glc3
6	*67.4* CH_2_	5.03 brd (12.6)		
		4.68 m		
Glc4 (1→4Xyl1)				
1	101.1 CH	4.94 d (7.0)	C: 4 Xyl1	H-4 Xyl1; H-3, 5 Glc4
2	*80.6* CH	4.77 t (8.3)	C: 1, 3 Glc4	H-4 Glc4
3	76.9 CH	4.31 t (8.3)	C: 2, 4 Glc4	H-1, 5 Glc4
4	70.7 CH	3.92 t (8.3)	C: 5, 6 Glc4	H-2 Glc4
5	77.5 CH	3.88 m		H-1 Glc4
6	61.8 CH_2_	4.35 brd (11.8)		
		4.03 dd (6.3; 11.8)	C: 5 Glc4	

^a^ Recorded at 176.04 MHz in C_5_D_5_N/D_2_O (4/1). ^b^ Bold: interglycosidic positions. ^c^ Italic: sulfate position. ^d^ Recorded at 700.13 MHz in C_5_D_5_N/D_2_O (4/1). Multiplicity by 1D TOCSY. The original spectra of **1** are provided in Figures S1–S7.

**Table 3:** The cytotoxic activities of glycosides **1**–**8**, desulfated psolusoside B and cisplatin (positive control) against human erythrocytes, human breast cancer cell lines MCF-7, T-47D and MDA-MB-231 and non-transformed MCF-10A cell line.

Glycosides	IC_50_, µM, Erythrocytes	Cytotoxicity, IC_50_ µM			
		MCF-10A	MCF-7	T-47D	MDA-MD-231
peronioside A (**1**)	30.80 ± 1.01	>50.0	>50.0	>50.0	>50.0
psolusoside A (**2**)	1.05 ± 0.12	>50.0	11.17 ± 0.40	9.52 ± 0.13	6.09 ± 0.87
psolusoside B (**3**)	74.57 ± 1.21	>50.0	>50.0	>50.0	>50.0
DS-psolusoside B	25.78 ± 2.55	46.22 ± 0.98	33.88 ± 1.64	31.20± 0.41	18.74 ± 1.65
psolusoside G (**4**)	2.70 ± 0.09	>50.0	11.43 ± 0.36	16.04 ± 1.17	10.99 ± 0.36
psolusoside I (**5**)	8.49 ± 0.49	>50.0	>50.0	19.56 ± 0.61	33.12 ± 0.85
psolusoside L (**6**)	1.83 ± 0.14	33.08 ± 0.29	13.74 ± 0.31	5.45 ± 0.04	6.39 ± 0.30
psolusoside N (**7**)	17.41 ± 0.41	>50.0	>50.0	>50.0	39.30 ± 2.89
psolusoside P (**8**)	7.77 ± 0.71	>50.0	>50.0	37.09 ± 0.83	33.29 ± 1.31
cisplatin	-	82.63 ± 4.62	70.39 ± 2.15	63.57 ± 1.94	66.23 ± 1.07

**Table 4:** Selectivity index (SI; a ratio of IC_50_ calculated for healthy and cancer cells) of glycosides **2** and **6** at 24, 48 and 72 h of exposition.

Glycosides	Time, h	Selectivity Index		
		MCF-7	T-47D	MDA-MB-231
psolusoside A (**2**)	24	4.47	5.25	8.21
	48	1.84	0.86	2.18
	72	0.59	0.76	2.09
psolusoside L (**6**)	24	2.41	6.07	5.18
	48	1.33	1.25	5.83
	72	0.66	0.91	4.56

## Data Availability

The raw data supporting the conclusions of this article will be made available by the authors on request.

## Supplementary Materials

The following are available online at https://www.mdpi.com/article/10.3390/md22070292/s1: Figures S1–S7: Original 2D and 1D NMR spectra of new glycoside **1**, Figure S8: (−) HR ESI MS and (−) ESI MS/MS spectra of **1**, Figures S9–S15: Original ^13^C NMR spectra of glycosides **2**–**8** with the signals assigned, Figure S16: Original ^13^C NMR spectrum of desulfated by C-2 Glc4 derivative of psolusoside B with the signals assigned, Figure S17: Photos of colonies of MCF-7, MDA-MB-231 and T-47D (C) cells, Figure S18: PLS QSAR model correlation plot, Figure S19: Intramolecular hydrogen bond networks in the glycosides, Videos 1–5: Migration of cancer cells.
