# Supplementary material for: The Composition of Triterpene Glycosides in the Sea Cucumber Psolus peronii: Anticancer Activity of the Glycosides against Three Human Breast Cancer Cell Lines and Quantitative Structure–Activity Relationships (QSAR)
Source: Mar Drugs. 2024 Jun 26;22(7):292. doi: 10.3390/md22070292 (PMC11278233; doi:10.3390/md22070292)
Supplement: Supplementary file 1 [file marinedrugs-22-00292-s001.zip › marinedrugs-3053153-supplementary.docx]

**Supplementary data content page**

Title: Composition of Triterpene glycosides in the Sea Cucumber *Psolus peronii*. Anticancer activity of individual glycosides against three human breast cancer cell lines. Quantitative structure-activity relationships (QSAR)

Authors: Alexandra Sergeevna Silchenko ^1,^*, Anatoly Ivanovich Kalinovsky ^1^, Sergey Anatolievich Avilov ^1^, Roman Sergeevich Popov ^1^, Ekaterina Alexandrovna Chingizova ^1^, Ekaterina Sergeevna Menchinskaya ^1^,
Elena Alexandrovna Zelepuga ^1^, Kseniya Mikhailovna Tabakmakher ^1^, Vadim Georgievich Stepanov ^2^ and Vladimir Ivanovich Kalinin ^1,^*

**Address:** ^1^ *G.B. Elyakov Pacific Institute of Bioorganic Chemistry, Far Eastern Branch of the Russian Academy of
Sciences, Pr. 100-letya Vladivostoka 159, 690022 Vladivostok, Russia*

^2^ *Kamchatka Branch of Pacific Institute of Geography, Far Eastern Branch of the Russian Academy of
Sciences, Partizanskaya st. 6, 683000 Petropavlovsk-Kamchatsky, Russia*

**Correspondence:** silchenko_als@piboc.dvo.ru (A.S.S.); kalininv@piboc.dvo.ru (V.I.K.);
Tel.: +7-423-231-1168

**Contents:**

Figure S1. The ^13^C NMR (176.04 MHz) spectrum of peronioside A (**1**) in C_5_D_5_N/D_2_O (4/1)

Figure S2. The ^1^Н NMR (700.13 MHz) spectrum of peronioside A (**1**) in C_5_D_5_N/D_2_O (4/1)

Figure S3. The COSY (700.13 MHz) spectrum of peronioside A (**1**) in C_5_D_5_N/D_2_O (4/1)

Figure S4. The HSQC (700.13 MHz) spectrum of peronioside A (**1**) in C_5_D_5_N/D_2_O (4/1)

Figure S5. The ROESY (700.13 MHz) spectrum of peronioside A (**1**) in C_5_D_5_N/D_2_O (4/1)

Figure S6. The HMBC (700.13 MHz) spectrum of peronioside A (**1**) in C_5_D_5_N/D_2_O (4/1)

Figure S7. 1D TOCSY (700.13 MHz) spectra of Xyl1, Glc2, Glc3, Glc4 of peronioside A (**1**) in C_5_D_5_N/D_2_O (4/1)

Figure S8. HRESI-MS (−) and ESI MS/MS spectra of peronioside A (**1**)

Figure S9. The ^13^C NMR (176.04 MHz) spectrum with the assigned signals of psolusoside A (**2**) in C_5_D_5_N/D_2_O

Figure S10. The ^13^C NMR (176.04 MHz) spectrum with the assigned signals of psolusoside B (**3**) in C_5_D_5_N/D_2_O

Figure S11. The ^13^C NMR (176.04 MHz) spectrum with the assigned signals of psolusoside G (**4**) in C_5_D_5_N/D_2_O

Figure S12. The ^13^C NMR (176.04 MHz) spectrum with the assigned signals of psolusoside I (**5**) in C_5_D_5_N/D_2_O

Figure S13. The ^13^C NMR (176.04 MHz) spectrum with the assigned signals of psolusoside L (**6**) in C_5_D_5_N/D_2_O

Figure S14. The ^13^C NMR (176.04 MHz) spectrum with the assigned signals of psolusoside N (**7**) in C_5_D_5_N/D_2_O

Figure S15. The ^13^C NMR (176.04 MHz) spectrum with the assigned signals of psolusoside P (**8**) in C_5_D_5_N/D_2_O

Figure S16. The ^13^C NMR (176.04 MHz) spectrum with the assigned signals of desulfated derivative by C-2 Glc4 of psolusoside B in C_5_D_5_N

Figure S17. The photos of colonies MCF-7, MDA-MB-231 and T-47D (C) cells in the control and under the treatment with 0.5, 1, 2, and 5 μM of psolusosides A (**2**) and L (**6**)

**T-47D**

**Psolusoside A (2), μM**

**Psolusoside L (6), μM**

**0.5	1	2	5**

**0.5	1	2	5**

Videos S1–S5. Migration of MDA-MB-231 cells in real time (15 min for each cycle) taken from live cell imaging system Juli Stage (NanoEntek, South Korea). 1 – control cells, 2 – cells under the treatment with 1 μM of psolusoside A (**2**), 3 – cells under the treatment with 2 μM of psolusoside A (**2**), 4 – cells under the treatment with 1 μM of psolusoside L (**6**), 5 – cells under the treatment with 2 μM of psolusoside L (**6**)

Figure S18. The PLS QSAR model correlation plot reflecting the relationship of predicted (pIC_50_) and experimental hemolytic activity. The PCA analysis of 87 selected calculated for 9 glycosides tested.

Figure S19. Intramolecular hydrogen bonds network of psolusosides I (**5**) (a) and DS-psolusoside B (b).

Figure S1. The ^13^C NMR (176.04 MHz) spectrum of peronioside A (**1**) in C_5_D_5_N/D_2_O (4/1)

Figure S2. The ^1^Н NMR (700.13 MHz) spectrum of peronioside A (**1**) in C_5_D_5_N/D_2_O (4/1)

Figure S3. The COSY (700.13 MHz) spectrum of peronioside A (**1**) in C_5_D_5_N/D_2_O (4/1)

Figure S4. The HSQC (700.13 MHz) spectrum of peronioside A (**1**) in C_5_D_5_N/D_2_O (4/1)

Figure S5. The ROESY (700.13 MHz) spectrum of peronioside A (**1**) in C_5_D_5_N/D_2_O (4/1)

Figure S6. The HMBC (700.13 MHz) spectrum of peronioside A (**1**) in C_5_D_5_N/D_2_O (4/1)

Figure S7. 1D TOCSY (700.13 MHz) spectra of Xyl1, Glc2, Glc3, Glc4 of peronioside A (**1**) in C_5_D_5_N/D_2_O (4/1)

Figure S8. HRESI-MS (−) and ESI MS/MS spectra of peronioside A (**1**)

Figure S9. The ^13^C NMR (176.04 MHz) spectrum with the assigned signals of psolusoside A (**2**) in C_5_D_5_N/D_2_O (4/1)

Figure S10. The ^13^C NMR (176.04 MHz) spectrum with the assigned signals of psolusoside B (**3**) in C_5_D_5_N/D_2_O (4/1)

Figure S11. The ^13^C NMR (176.04 MHz) spectrum with the assigned signals of psolusoside G (**4**) in C_5_D_5_N/D_2_O (4/1)

Figure S12. The ^13^C NMR (176.04 MHz) spectrum with the assigned signals of psolusoside I (**5**) in C_5_D_5_N/D_2_O (4/1)

Figure S13. The ^13^C NMR (176.04 MHz) spectrum with the assigned signals of psolusoside L (**6**) in C_5_D_5_N/D_2_O (4/1)

Figure S14. The ^13^C NMR (176.04 MHz) spectrum with the assigned signals of psolusoside N (**7**) in C_5_D_5_N/D_2_O (4/1)

Figure S15. The ^13^C NMR (176.04 MHz) spectrum with the assigned signals of psolusoside P (**8**) in C_5_D_5_N/D_2_O (4/1)

DS-psolusoside B

Figure S16. The ^13^C NMR (176.04 MHz) spectrum with the assigned signals of desulfated by C-2 Glc4 derivative of psolusoside B in C_5_D_5_N

Figure S17. The photos of colonies MCF-7, MDA-MB-231 and T-47D (C) cells in the control and under the treatment with 0.5, 1, 2, and 5 μM of psolusosides A (**2**) and L (**6**).

**T-47D**

**Psolusoside A (2), μM**

**Psolusoside L (6), μM**

**0.5	1	2	5**

**0.5	1	2	5**

Videos S1–S5. Migration of MDA-MB-231 cells in real time (15 min for each cycle) taken from live cell imaging system Juli Stage (NanoEntek, South Korea). 1 – control cells, 2 – cells under the treatment with 1 μM of psolusoside A (**2**), 3 – cells under the treatment with 2 μM of psolusoside A (**2**), 4 – cells under the treatment with 1 μM of psolusoside L (**6**), 5 – cells under the treatment with 2 μM of psolusoside L (**6**).

Figure S18. The PLS QSAR model correlation plot reflecting the relationship of predicted (pIC_50_) and experimental hemolytic activity. The PCA analysis of 87 selected calculated for 9 glycosides tested.

Figure S19. Intramolecular hydrogen bonds network of psolusosides I (5) (a) and DS-psolusoside B (b). Molecular structures are presented as sticks, HBs аre presented as blue dashed line.
